# Verification of Cundurango

**Published:** 1895-06

**Authors:** U. Goullon

**Affiliations:** Weimar


### VERIFICATION OF CUNDURANGO.

DR. U. GOULLON, WEIMAR.

An old farmer suffers from an ulcer on his lip. The lower lip is deeply eroded, with irregular edges surrounding a deep ulcer, its base filled with foul, purulent matter. It began with small blisters, which discharged and covered themselves with crusts. These fell off, but it always reappeared, till now it forms a malignant ulcer. Cundurango, 2d dec., thrice daily, two drops. After two weeks the ulcer was gone, only a thin crust yet covered its former place, which without further treatment soon left a healthy skin.—*Populäre Zeitsch, f. H.* 12, 91.

